# Discrete roles of RNA helicases in human male germline and spermatogenesis

**DOI:** 10.1007/s13353-020-00558-4

**Published:** 2020-04-24

**Authors:** Katarzyna Tutak, Natalia Rozwadowska

**Affiliations:** Institute of Human Genetics, Polish Academy of Science, Strzeszynska 32, 60-479 Poznan, Poland

**Keywords:** RNA helicases, DEAD/DEAH-box, Spermatogenesis, Infertility, Germline

## Abstract

RNA helicases are known from their ability to bind and unwind double-stranded RNA initiating RNA processing events. These evolutionary conserved RNA binding proteins are broadly expressed in a variety of tissues; however, we can distinguish those, which represent tissue-specific expression pattern and play unique roles in certain cell lineages. For instance, some RNA helicases mediate transcriptomic changes triggering cell differentiation which results in specification and establishment of germline in a developing embryo. Others act as safeguards responsible for maintenance of DNA integrity in germ cell. In this article, we focus on selected DEAD/DEAH-box RNA helicases involved in germline development and spermatogenesis presenting their diverse functions and implications for male fertility.

## Introduction

**Fig. 1:**
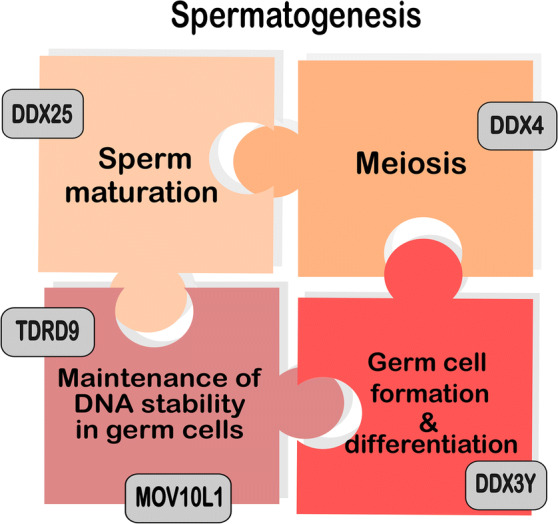
Involvement of selected RNA helicases in processes leading to spermatogenesis

The large family of RNA helicases orchestrates gene expression by contributing to RNA metabolism during a whole life span of these molecules. These enzymes are classified as a vast group of highly conserved proteins characterized by their roles in rearrangement of RNA secondary structure and facilitation of RNA-protein interactions in an ATP-dependent manner. The ability of these enzymes to unwind RNA initiates cascade of posttranscriptional regulation events such as splicing, translation initiation and mRNA decay (Bourgeois et al. 2016). Additionally, to their roles in RNA metabolism, these proteins are engaged in a variety of different biochemical activities described in detail by other authors (Putnam and Jankowsky 2013). In this mini-review, we aim to focus on unique functions of selected DEAD/DEAH-box RNA helicases with an emphasis on human male germline development and spermatogenesis (Table 1, Fig. 1).

**Table 1:** Functions of selected RNA helicases in germ cells and spermatogenesis

RNA helicase	Main molecular process	Function in germ cells/spermatogenesis	References
DDX3Y/DBY	Control over expression of mitotic cyclins (*Drosophila)* Activation of germline-specific developmental genes Regulation of differentiation towards germ cell lineage	Regulation of mitotic progression (*Drosophila*) Germ cells generation and maintenance	Kotov et al. (2017) Ramathal et al. (2015)
DDX25/GRTH	Formation of ribonucleoprotein complexes Regulation of translation Export RNAs to chromatoid bodies Chromatin remodeling Regulation of *Tp2* transcripts	Spermatid elongation Sperm maturation	Tsai-Morris et al. (2010) Yang et al. (2015) Kavarthapu et al. (2019)
DDX4/VASA	Regulation of expression of early and late germ cell markers piRNA pathway	Promotion of meiotic progression in human germ cells Development of germline and homeostasis maintenance (*Drosophila*)	Medrano et al. (2012) Sugimoto et al. (2009) Durdevic and Ephrussi (2019)
TDRD9	piRNA-mediated retrotransposon silencing	Maintenance of DNA stability in germ cells	Wenda et al. (2017)
MOV10L1			Frost et al. (2010) Vourekas et al. (2015)

## RNA helicases: general overview

The majority of RNA helicases belong to two main superfamilies (SF1 and SF2) comprised of subfamilies divided based on protein primary sequence variation. In humans, among SF2 group, there are two main subfamilies distinguished: the DEAD-box (DDX) helicases containing Asp-Glu-Ala-Asp (DEAD) motif represented by 42 members and DEAH-box (DHX motif: Asp-Glu-Ala-His) helicases with 16 members (Fairman-Williams et al. 2010). The most characteristic feature of RNA helicases in SF1 and SF2 families is their conserved core comprising two tandemly repeated RecA domains. These domains (named after the bacterial homologous recombination protein RecA) are responsible for nucleic acid and ATP binding and hydrolysis. Most of RNA helicases on amino or/and carboxyl termini possess an RNA or protein recognition motif which enhances the specificity of their interactors and targets. Common feature for almost all RNA helicases is the ability to bind RNA, nucleotides, ATP, and proteins. It is well described that the interplay between all these molecules affects both their conformation and function. Some helicases can also act as scaffolds for building ribonucleoprotein complexes (Putnam and Jankowsky 2013). However, one of the highly investigated and well described functions of RNA helicases is their ability to unwind RNA. Based on the catalytic mechanism, two main groups of helicases can be distinguished. One group encompasses so called processive RNA helicases, which mediate the unwinding of double-stranded RNA in the canonical ATP-dependent manner by binding and translocating along RNA duplexes. The second group containing DEAD-box helicases that resolve RNA duplexes in the non-processive way by local strand separation facilitated by ATP hydrolysis (Bourgeois et al. 2016; Putnam and Jankowsky 2013).

## Involvement of DEAD-box helicases in germ cell lineage development and spermatogenesis

DEAD-box RNA helicases influence cell fate by contributing to RNA metabolism (Bourgeois et al. 2016). Transcriptomic changes triggered by RNA helicases initiate differentiation processes and result in a development of cell lineages (Medrano et al. 2012; Ramathal et al. 2015) Below, we describe examples of DEAD-box helicases: DDX3Y, DDX25, and DDX4/VASA in contexts of germline generation, spermatogenesis, and male infertility.

### DDX3Y/DBY

Gene encoding DEAD-Box Helicase 3 Y-linked protein (DDX3Y or DBY) is located in azoospermia factor (AZF) Y-chromosomal locus which is associated with male fertility. The data presented by Kotov et al. (2017) indicates that Belle, homolog of DDX3Y in *Drosophila*, plays an important role in mitotic progression by controlling the expression of mitotic cyclins A and B in *Drosophila* testes. In addition, Belle regulates maintenance and cell division of germ stem cells (GSC). In humans, DDX3Y is expressed in germ cells at the premeiotic stage of spermatogenesis and mutations in this gene are associated with Sertoli cell-only (SCO) syndrome leading to azoospermia and infertility (Foresta et al. 2000). Studies performed by Ramathal et al. (2015) in a xenotransplantation model demonstrate an increased expression of germline-specific genes (*PRDM14*, *NANOG*, *LIN28A*) and a significant progression in differentiation of AZFa-deleted human-induced pluripotent stem cells (iPSCs) into germ cell-like cells post restoration of DDX3Y protein. Presence of DDX3Y evoked higher expression of transcriptional and translational regulator genes such as *DDX21*, *SF3A1*, *RPRD2*, and a group of zinc-finger proteins, which might suggest an initiation of global changes in transcriptome activating germ cell differentiation. Taken together, these findings emphasize a significant contribution of DDX3Y helicase in the germ cell’s fate determination.

### DDX25

DEAD-box helicase 25 (DDX25) also described as gonadotropin-regulated testicular RNA helicase (GRTH) is present in meiotic spermatocytes, round spermatids, and Leydig cells and its expression is controlled by hormonal stimulation via gonadotropin/androgen regulation. DDX25 is a multifunctional protein, which facilities several molecular events crucial for spermatogenesis completion. DDX25 binds to various mRNAs and determines their fate by forming ribonucleoprotein complexes, mediating export of transcripts from nucleus to cytoplasmic chromatoid bodies of spermatids for their further translation during spermatogenesis (Tsai-Morris et al. 2010). Studies performed by Yang et al. (2015) emphasize the significance of DDX25 in male gamete formation by demonstrating that this helicase contains motif responsible for recognition of transcripts encoding transition protein 2 (Tp2) required for spermatid elongation. In addition, a newly identified mutation in *DDX25* gene found in azoospermic men results in phosphorylation disruption of the helicase, which interrupts chromatin remodeling and contributes to germ cells apoptosis. This finding underlines a significant influence of DDX25 protein on spermatogenesis completion (Kavarthapu et al. 2019).

### DDX4/VASA

*DDX4* gene also called *VASA* encodes DEAD-box RNA helicase 4. Homologs of this protein are present among various invertebrate and vertebrate species including *C. elegans*, *D. melanogaster*, *D. rerio*, *X. laevis* and rodents. Despite discrepancies in functions between species, this cytoplasmic protein seems to be crucial for germ cell development and fertility. In mammals, this helicase starts to be expressed in migratory primordial germ cells and continues to be present throughout gametogenesis. The highest expression is observed in spermatocytes and mature oocytes (Castrillon et al. 2000). Studies conducted by Medrano et al. (2012) indicate that ectopic expression of VASA in pluripotent stem cell lines leads to the upregulation of early (*BLIMP1*, *GDF3*, *GCNF*) and late germ cell markers (*SCP3*, *ZP4*, *GDF9*) and increases the percentage of cells positive for postmeiotic marker (ACROSIN) after differentiation towards germ cell-like cells. This leads to the conclusion that VASA plays significant role in promotion of meiotic progression in human germ cells derived from pluripotent stem cells. Studies of male patients with azoospermia or oligozoospermia imply that hypermethylation status of *VASA* promoter may lead to maturation arrest phenotype in testis and results in infertility (Sugimoto et al. 2009). Yu et al. (2016) analyzed semen samples from non-obstructive azoospermic (NOA) patients and indicate that noninvasive evaluation of *VASA* transcripts in cell-free seminal mRNAs may become more accurate way to improve identification of specific group of NOA and discrimination SCO. In conclusion, restricted expression to germinal lineage and significant involvement in germ cell development make this protein an important and vastly studied marker in contexts of infertility and spermatogenic cell differentiation.

## RNA helicases as safeguards of maintaining the DNA stability in germline

Increasing number of evidences supports the statement that PIWI/piRNA pathway and its implications for transposon control are crucial for homeostasis in germline. The control over mobile genetic elements seems to be essential for successful male germline formation and production of functional gametes. For instance, recently published studies in *Drosophila* concerning previously mentioned RNA helicase Vasa illustrates Vasa-dependent transposon control. It is shown that under Vasa depletion conditions, oogenesis is not completed possibly due to upregulation of selfish genetic elements which cause DNA damage-induced oogenesis arrest (Durdevic and Ephrussi 2019). Article published by Hempfling et al. (2017) shows that among human testisbiopsies representing normal and abnormal spermatogenesis, piRNA key pathway components such as PIWIL proteins and HENMT1 (responsible for methylation of piRNA 3′ end) present different expression patterns. In infertile man, expression on these genes was significantly lower which resulted in elevated level of *LINE-1*. This indicates that disturbances in piRNA pathway abolish transposon control and may lead to spermatogenesis impairment.

### TDRD9

Another example of RNA helicase involved in piRNA-mediated transposon silencing is TDRD9 (Tudor domain containing 9). This protein comprises DEAH-box domain responsible for ATP-dependent RNA unwinding and Tudor domain which recognizes methylated arginine and lysine residues and mediates macromolecular complex assembly. In mice, TDRD9 helicase forms complex with MIWI2 protein which is involved in piRNA-mediated LINE-1 retrotransposons silencing. The experimental mouse model with knock-in allele comprising mutations within *Tdrd9* ATPase motif represents male sterility, indicating the crucial role of ATPase activity of TDRD9 helicase in mice spermatogenesis (Wenda et al. 2017). Concerning human spermatogenesis, Arafat et al. (2017) analyzed samples from a group of non-obstructive azoospermic men and identified mutations resulting in frameshift and exon skipping in *TDRD9* transcripts. Authors claimed that presented mutations were responsible for maturation arrest which led to infertility.

### MOV10L1

MOV10L1 (Mov10 like RISC complex RNA helicase) is involved in genetic stability maintenance in germ cells. In mice, homologous protein is shown to be a key regulator of piRNA-directed LINE-1 retrotransposon silencing in male germline (Frost et al. 2010). In humans, the underlying mechanism of this regulation was presented by Vourekas et al. (2015) showing that MOV10L1 specifically binds to piRNA precursors, which initiates their processing. Subsequently, piRNA intermediate fragments are loaded to PIWI proteins, which lead to the suppression of retrotransposon activation. RNA helicase MOV10L1 is abundant in testis, and its expression begins in prenatal gonocytes and ends in postmeiotic spermatids. In addition, single-nucleotide polymorphisms in *MOV10L1* gene were identified in infertile men and associated with spermatocyte maturation arrest resulting in azoospermia (Sarkardeh et al. 2014).

## Conclusions

RNA helicases are evolutionary conserved proteins present in a broad range of species. Growing number of evidence emphasizes the fact that the role of these proteins in developmental processes is crucial. RNA helicases play distinct roles in promoting germline establishment and maintenance and gametogenesis. They act as safeguards of germline DNA stability and influence changes in transcriptome by contributing to meiotic progression and sperm maturation. Importance of these proteins is supported by data indicating that several mutations in RNA helicases genes are associated with male infertility. Relevance in spermatogenesis combined with mutations occurrence among male population implies that RNA helicases may serve as potential genetic markers improving diagnostics of infertile men. Despite the demonstrated significance in male gamete formation, the exact mechanisms, functions, and pathways involving RNA helicases remain to be elucidated and demand further investigation.
